# Cytokine Changes following Acute Ethanol Intoxication in Healthy Men: A Crossover Study

**DOI:** 10.1155/2016/3758590

**Published:** 2016-12-20

**Authors:** Sudan Prasad Neupane, Andreas Skulberg, Knut Ragnvald Skulberg, Hans Christian D. Aass, Jørgen G. Bramness

**Affiliations:** ^1^Norwegian Center for Addiction Research (SERAF), University of Oslo, Oslo, Norway; ^2^Norwegian National Advisory Unit on Concurrent Substance Abuse and Mental Health Disorders, Innlandet Hospital Trust, Brumunddal, Norway; ^3^Primo A/S, Larkollen, Norway; ^4^Hedmark University of Applied Sciences, Elverum, Norway; ^5^Department of Medical Biochemistry, Oslo University Hospital, Oslo, Norway

## Abstract

Alcohol is a known modulator of the innate immune system. Owing to the absence of human studies, alcohol's effect on circulating cytokine profile remains unclear. We investigated the effect of acute high dose alcohol consumption on systemic cytokine release. After an overnight fasting, alcohol-experienced healthy male volunteers (*N* = 20) aged 25–45 years were given oral ethanol in the form of vodka (4.28 mL/kg) which they drank over a period of 30 minutes reaching peak blood alcohol concentration of 0.12% (SD 0.028). Blood samples were obtained prior to alcohol intake as well as 2, 7, and 12 hours thereafter. Serum levels of the inflammatory cytokines IL-1*β*, IL-1Ra, IL-6, IL-10, IL-17, IFN-*γ*, MCP-1, and TNF-*α* were determined by the multibead-based assay. Baseline cytokine levels were not related to BMI, hepatic parameters, electrolytes, glucose, or morning cortisol levels. Within 2 hours of alcohol intake, levels of IL-1Ra were elevated and remained so throughout the assessment period (*p* for trend = 0.015). In contrast, the levels of the chemokine MCP-1 dropped acutely followed by steadily increasing levels during the observation period (*p* < 0.001). The impact of sustained elevated levels of MCP-1 even after the clearance of blood alcohol content deserves attention.

## 1. Introduction

Alcohol is a known modulator of the immune system affecting innate as well as adaptive arms of the host immune response. Excessive and chronic heavy drinking, as typified in alcohol use disorder (AUD), induces systemic and CNS inflammation [1] which contribute to the development of a number of alcohol-attributable chronic diseases and conditions [2]. One widely proposed mechanism for innate immune response in chronic heavy alcohol consumption involves alcohol-induced changes in the composition of gut microbiome [3] and compromised gut wall integrity [4] allowing bacterial products such as lipopolysaccharide (LPS) to “leak” into systemic circulation which promotes secretion of proinflammatory cytokines including tumor necrosis factor-alpha (TNF-*α*) and interleukin- (IL-) 1*β* through Toll-like receptor mediated activation of transcription factors, such as nuclear factor-*κ*B [5, 6]. High dose alcohol exposure can induce neuroimmune signaling even after a single alcohol binge [7, 8] and immune stimuli such as LPS may not be necessary for inducing these changes [9, 10].

A recent study [11] demonstrated increased hippocampal IL-10 content in adult rats one hour after a single intoxicating intragastric dose of ethanol (5 g/kg). In mice pretreated with ethanol, Qin and colleagues found that LPS-induced production of TNF-*α*, IL-1*β*, and monocyte chemoattractant protein 1 (MCP-1, also known as CCL_2_) were elevated in the liver, serum, and brain [12]. Increases in serum cytokine levels subsided by 9 hours with clearance of blood alcohol content. Importantly, the same group discovered that a single immune stimulus was sufficient to activate brain microglia to produce chronically elevated inflammatory factors in rodent models [13]. These lines of evidence suggest that occasional ethanol intoxication can have far-reaching consequences through neuroimmune modulation. However, the nature of those consequences is unclear because of the paucity of experimental alcohol studies in humans.

Generally, acute alcohol exposure (usually defined as less than 24 hrs) favors anti-inflammatory response and chronic alcohol consumption favors proinflammatory cytokine release [14, 15]. For instance, healthy men and women 20 minutes after binge alcohol consumption (0.9 g/kg in men and 0.8 g/kg in women) were found to have elevated blood leukocytes, monocytes, natural killer cells, and LPS-induced TNF-*α* production which switched towards anti-inflammatory direction after 2 hours [7]. The dynamic of the immune response, thus, seems to be more complex and depends on the dose as well as time duration since alcohol intake. This study aimed to further elucidate the effects of binge alcohol intoxication in physiologically relevant conditions in humans by assessing changes in serum cytokine levels up to 12 hours after drinking.

## 2. Materials and Methods

### 2.1. Study Participants

Healthy volunteers were recruited through an open advertisement in the Correctional Service of Norway Staff Academy in Oslo. Inclusion criteria were male gender, age 20–45 years, and Caucasian origin who admitted having experiences of high dose alcohol drinking sometime in the past. Exclusion criteria were significant medical illness, alcohol or other substance use disorders, and metabolic disorders.

Volunteers interested in participating in the study were invited to be screened for AUD as well as physical and psychological health. Demographic information was recorded and biochemical parameters were determined by venous blood drawn after an overnight fasting. Participants had no active inflammation at the time of experiment indicated by prealcohol CRP levels < 20. AUD screening was done by using the Alcohol Use Disorder Identification Test (AUDIT) [16]. All individuals scored below 15 in the AUDIT scale, consistent with being nondependent on alcohol. This was confirmed by measuring serum carbohydrate deficient transferrin (CDT) value at baseline which was <1.7% in all participants.

### 2.2. Experimental Setup

The experiment was a part of a drug trial on ethanol metabolism based on a double-blind crossover design investigating the impact of phosphate supplement on ethanol metabolism. Participants showed up at the experiment location at 07:00 in the morning following an overnight fasting. Baseline blood samples were taken 15 minutes after arrival and then a light breakfast was served. After thirty minutes participants were served oral ethanol in the form of vodka (38% by volume) in a dose of 4.28 mL/kg which they drank over a period of 30 minutes reaching peak blood alcohol concentration of 0.12% (SD 0.028). Participants were allowed standard meals and indoor activities throughout experiment.

In addition to prealcohol samples (T_1_) obtained in the fasting state, venous blood samples were obtained from the cubital region (cephalic or median cubital vein) a total of 10 times during 12 hours of observation. Sampling at 2 (T_2_), 7 (T_3_), and 12 hours (T_4_) after alcohol intake was used for cytokine assays. Intervention and control solutions used in the experiment contained elemental phosphate and dextrose based drinks which are not known to impact cellular immune response. The experiment was repeated after one week swapping the intervention and control group participants making individuals their own controls.

### 2.3. Background Variables

Background variables were registered under the categories somatic, psychological, and anthropometric variables. History of specific organ damage, chronic inflammatory conditions, and atopy was inquired. Recent use of drugs of abuse and alcohol use-related adverse consequences, such as subjective dose required for intoxication, and vomiting were also recorded. Body mass index was calculated as weight (Kg)/height (m)^2^. Routine biochemistry was done on all participants 10 days prior to the first experiment day to determine eligibility and to assess baseline values.

### 2.4. Laboratory Analyses

Routine biochemical tests for screening purpose included blood hemoglobin, C-reactive protein, mean corpuscular volume and hemoglobin, serum glucose, CDT, gammaglutamyl transferase (GGT), creatinine, phosphorus, and alanine aminotransferase enzyme assays. Blood alcohol concentrations were measured at ten times during the observation and supplemented by six breathalyzer readings.

Cytokine measurements were performed using a Luminex IS 100 instrument (Bio-Rad, Hercules, California, USA), equipped with the Bio-Plex Manager software (version 6.0.1). A custom-made kit was purchased to screen all samples consisting of the cytokines IL-1*β*, IL-1Ra, IL-6, IL-10, IL-17, IFN-*γ*, MCP-1, and TNF-*α*. Individual sets of samples from one participant were run in the same assay but kept blinded. All samples were thawed on ice, vortexed, and then spun down at 10.000 ×g for 10 min at 4°C, before dilution (1 + 4) and further processing. The assay was performed according to the manufacturer's instructions, except for the additional standard point to enable low level detection. All cytokine assays were performed in duplicate. Longitudinal control was used to determine inter-% CV to evaluate assay performance of the assay (IL-1*β* (5.6), IL-Ra (4.1), IL-6 (5.5), IL-10 (5.3), IL-17 (6.2), IFN-*γ* (7.1), MCP-1 (5.4), and TNF-*α* (5.3)). The results for the serum samples that were found to have levels lower than the detectable limit (set at 0) were IL-1*β* (22%), IL-1Ra (0%), IL-6 (60%), IL-10 (69%), IL-17 (84%), IFN-*γ* (92%), MCP-1 (8%), and TNF-*α* (76%). This number of missing data was not different across the 4 time points of observation. Therefore, we are reporting only IL-1*β*, IL-1Ra, and MCP-1 in the results.

### 2.5. Statistical Analyses

The statistical analysis was performed using IBM SPSS Statistics for Windows version 22 (IBM Corp., Armonk, NY). Cytokine data had skewed distribution and therefore we chose nonparametric tests (Mann–Whitney *U* test, Kruskal–Wallis test, and Spearman's rho) for comparisons. Results were considered significant when *p* < 0.05. Pairwise comparisons were made for cytokine changes over time using general linear models with Bonferroni corrections when needed. Relative changes between the levels of each cytokines were compared using correlation matrix on respective first principal components of the principal component analysis [17].

### 2.6. Ethics

The study protocol was approved by the Norwegian Regional Ethics Committee (REK case ref. 2013/1563). Prior to inclusion, written informed consent was obtained from all participants. Participants were fully entitled to withdraw their consent at any time during the study. They received bank transfer amounting Norwegian Kroner 2000/day in compensation to the time incurred for the experiment and taxi fare was paid to return home after the experiment. All participants were ensured as part of the project leader's membership with the Norwegian Drug Liability Association (ref. 5041916/1) which covers eventualities in connection with a clinical drug trial.

## 3. Results

The participant characteristics including demographics, AUDIT scores, biochemical parameters, and allergic history are given in Table 1. The prealcohol (T_1_) and subsequent (T_2_, T_3_, and T_4_) levels of IL-1*β*, IL-1Ra, and MCP-1 did not vary between the two experimental days. T_1_ levels of IL-1Ra and MCP-1 on experimental days 1 and 2 were highly correlated (*ρ* = 0.91; *p* < 0.001 and *ρ* = 0.82; *p* < 0.001, resp.). The variations in the cytokine levels across the observation period were represented by the respective principal components found in the principal component analysis. The first principal component (FPC1) of IL-1Ra and MCP-1 explained more than 90% of all variations in these cytokine values over time. The FCP1s for IL-1Ra and MCP-1 were highly correlated (*ρ* = 0.64, *p* < 0.001). Phosphate intervention given as part of the drug trial had no effect on the cytokine levels on either day (*p* > 0.05 for all test results). Among the analytes included, T_1_ levels of IL1Ra correlated with baseline MCV values (*ρ* = 0.66, *p* = 0.002) and T_1_ levels of MCP-1 correlated with serum GGT levels (*ρ* = 0.522, *p* = 0.02). The cytokines were unrelated to age, BMI, or AUDIT scores (*p* > 0.05 for all correlation analyses).

**Figure 1:**
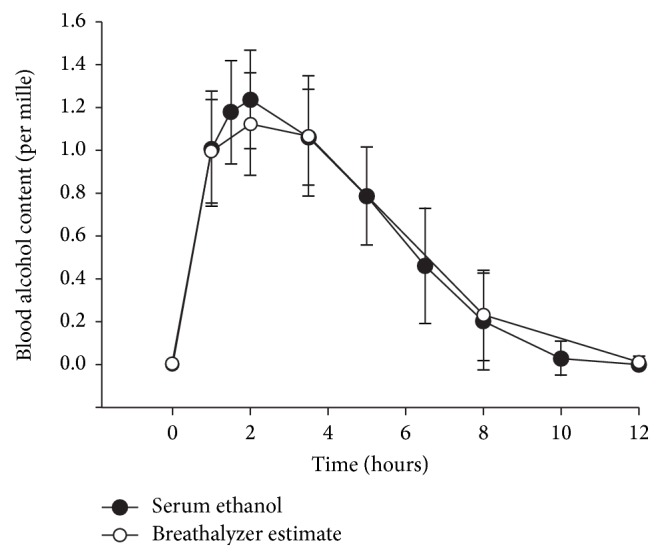
Elimination curve of ethanol following binge drinking of vodka amounting 4.28 mL/kg body weight as assessed by blood ethanol content and breathalyzer estimate.

**Figure 2:**
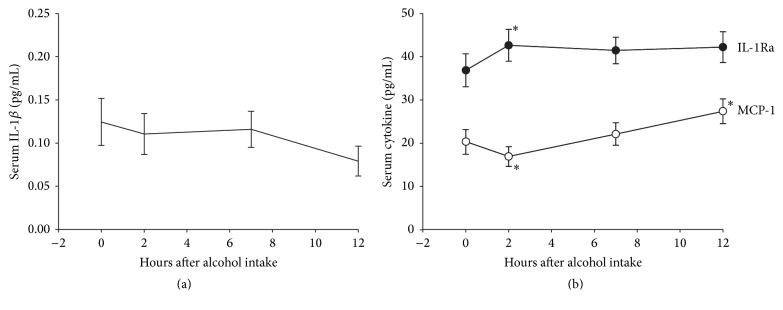
(a) Serum levels (mean and standard error of mean) of IL-1*β* at baseline and 2, 7, and 12 hours after binge drinking of alcohol. No statistically significant trend or differences between observations were found. (b) Serum levels (mean and standard error of mean) of IL-1Ra and MCP-1 at baseline and 2, 7, and 12 hours after binge drinking of alcohol. Statistically significant changes from prebinge values were observed at T_2_ for IL-1Ra and at T_2_ and T_4_ for MCP-1 and are indicated by an *∗*. Trend statistics were significant for both cytokines.

Blood alcohol content measured directly as well as breathalyzer estimates showed normal peak and elimination curve (Figure 1). IL-1*β* levels did not change over the observation period (*p* = 0.077) (Figure 2(a)). However, IL-1Ra and MCP-1 levels changed significantly (*p* = 0.015 and *p* < 0.001, resp.). Within 2 hours of alcohol intake, levels of IL-1Ra were elevated and remained so throughout the assessment period. The levels of MCP-1 however dropped acutely followed by steadily increasing levels during the observation period which remained higher than baseline (*p* = 0.025) at the 12th hour (Figure 2(b)).

## 4. Discussion

We investigated in a cohort of healthy men the changes in serum levels of inflammatory cytokines following alcohol intake in a euphoric dose. Results demonstrate an early cytokine response followed by changes that may last beyond complete elimination of ethanol from circulation. This finding extends earlier suggestions stemming from in vitro and preclinical works that high dose ethanol can trigger long-lasting innate immune responses [12, 18]. Here we show in vivo changes in cytokine levels following alcohol intoxication which seems to be independent of an ongoing traumatic injury or infection. These findings are important because alcohol-induced changes in cytokine pathways with and without an immune challenge can manifest in a range of mood and behavioral disorders, particularly in the context of chronic heavy drinking [19].

Findings from this study are in line with a handful of human studies that have been previously conducted examining direct effects of alcohol intoxication on cytokine response. Not only binge does drinking in healthy individuals rapidly increase serum LPS through compromised gut wall integrity but also the same dose of LPS ex vivo can induce production of proinflammatory cytokines, TNF-*α*, IL-6, and MCP-1 [8]. We demonstrate postalcohol induction of inflammatory cytokine MCP-1 in vivo within hours following an apparently acute fall in value that corresponds with opposing changes in IL-1Ra which has an anti-inflammatory effect. Indeed, acute increases in the levels of another anti-inflammatory cytokine IL-10 have been demonstrated also in the brain of rats fed with high dose ethanol [11]. The changes in the levels of IL-1Ra may not be coincidental as it is essential for alcohol drinking preference typical of AUD [20]. Also, our data shows an association between GGT, a measure of alcohol-induced liver injury, and MCP-1 levels. Although higher values of cytokines are not expected in normal physiology, the detectable ranges of IL-1*β*, IL-1Ra, and MCP-1 are among the most commonly reported cytokines that could be important candidate immune mediators in alcohol-induced tissue injury and long-term psychiatric and behavioral outcomes. Clearly, MCP-1 expression levels have been found to be uniquely related to blood alcohol content [21]. MCP-1 is responsible for recruitment and activation of monocytes in the process of alcohol liver disease [22]. In specific brain areas of rodents that regulate drinking behavior, MCP-1 has been found colocalized and overexpressed with Toll-like receptor 4, a potent sensor of LPS [23, 24]. This supports the role of MCP-1 in alcohol use disorder.

In the context of heavy drinking, LPS-induced production of the cytokines TNF-*α*, MCP-1, and IL-1*β* are potentiated [12]. A high degree of correlation between the MCP-1, a proinflammatory chemokine, and IL-1Ra, an anti-inflammatory cytokine, on both experimental days indicates that, under physiological conditions, a balanced pro- and anti-inflammatory state is maintained. During the first hours of alcohol intake, rise in the level of IL-1Ra was accompanied by a fall in MCP-1 levels. The balance persisted over the course of the observation period in which changes in the cytokine levels were found. Since prealcohol cytokine levels were similar on the two experimental days our findings do not support a spillover effect of alcohol sensitization of immunologically active cells to produce higher levels of cytokines in the context of occasional heavy drinking.

A limitation to this study is that cytokine changes were not compared against a normal diurnal variation. Although cytokine profile has been found to vary according to the sampling hour of the day, the difference is less marked in serum samples compared to the plasma [25]. Some inflammatory cytokines, but not anti-inflammatory cytokines, seem to increase slightly with diurnal reduction in cortisol levels [25, 26]. Also, several animal studies have indicated that ethanol administration in itself can induce production of inflammatory cytokines and chemokines in the brain as well as in the periphery [27, 28]. With the present data, we are unable to comment on whether the subtle changes observed in serum translate into CNS changes. The study samples, like most other experimental human studies, are too small to compare between different variables and subgroups of individuals, such as the interaction of atopy in alcohol-related immune changes. Larger samples are required to increase power and to limit type II error. Alcohol-immune interaction in the face of concurrent immune-inflammatory conditions should be investigated in future studies. Including only healthy male subjects in the middle age avoided gender- and ageing-related differences in cytokines. Although the findings provide important insight into occasional heavy drinking, the response of immune cells potentially sensitized after prolonged alcohol exposure needs to be examined in a clinical sample.

In summary, this study demonstrates that binge drinking provokes an early cytokine response favoring anti-inflammatory state, as indicated by elevated IL-1Ra levels, followed by a gradual change towards the baseline levels, which are not attained even when ethanol is fully eliminated from the blood. On the contrary, the levels of the proinflammatory chemokine MCP-1 increased after initial drop and remained higher than baseline levels at 12-hour follow-up. Allostatic changes in neuroimmune parameters upon repeated alcohol challenge may underlie a range of alcohol-related diseases and injuries.

## Figures and Tables

**Table 1:** Participants' baseline characteristics and biochemical parameters assessed 10 days prior to the experiment.

Participant	Age (years)	Body mass index	AUDIT score (total)	Carbohydrate deficient transferrin (%)	B-Hemoglobin (g/dL)	Mean corpuscular volume (fL)	Mean corpuscular hemoglobin (pg)	Serum alanine aminotransferase (U/L)	Serum cortisol (nmnol/L)	Random blood glucose (mmol/L)	Serum creatinine (*µ*mol/L)	Serum gamma-GT (U/L)	C-reactive protein	History of atopy
1	29	24.69	15.00	0.7	15	90	31	33	456	4.8	86	27	0.5	Yes
2	26	26.04^*∗*^	13.00	1.3	15.1	94	34^*∗*^	28	374	4.9	82	31	0.5	No
3	26	23.08	8.00	0.4	15	87	31	17	610	5	71	5	0.5	Yes
4	25	22.35	5.00	0.8	15.8	87	32	27	616	4.8	74	18	2	No
5	26	22.95	7.00	1.3	16.3	88	30	38	676	5	87	35	0.5	No
6	26	23.77	9.00	0.7	15.6	91	30	26	471	4.3	81	18	0.5	No
7	27	24.69	12.00	0.7	15.9	88	31	30	574	4.5	78	34	1	Yes
8	29	25.43^*∗*^	10.00	0.6	15.2	87	30	43	495	5.3	83	38	0.5	No
9	29	28.80^*∗*^	2.00	0.9	15.3	83	29	56	438	4.8	76	51	1	Yes
10	45	24.10		0.7	15	85	30	31	528	4.1	103	16	0.5	
11	26	24.76	10.00	1.0	16.5	90	31	35	643	4.8	101	19	0.5	Yes
12	29	23.25	14.00	0.7	15.8	85	30	24	841	5.4	95	20	0.5	No
13	42	28.09^*∗*^	8.00	0.4	16.4	87	30	50	360	4.8	81	41	0.5	No
14	28	27.68^*∗*^	5.00	0.7	16.1	84	28	21	523	4	70	38	0.5	Yes
15	28	23.63	8.00	0.8	15.1	91	31	25	623	4.8	101	22	0.5	Yes
16	27	24.71	8.00	0.6	15.5	84	29	33	455	4.9	82	18	2	Yes
17	30	21.91	10.00	0.8	14	92	32	35	555	4	82	19	16^*∗*^	No
18	25	26.60^*∗*^	10.00	0.6	15.7	96	32	34	617	4.2	109^*∗*^	16	0.5	No
19	29	30.79^*∗*^	10.00	0.8	14.7	96	34^*∗*^	45	570	4.8	82	52	4	Yes
20	25	29.40^*∗*^	10.00	0.4	15.8	88	31	31	711	4.6	91	21	0.5	No

^*∗*^Values outside reference range for healthy men.

## References

[1] Wang H. J., Zakhari S., Jung M. K. (2010). Alcohol, inflammation, and gut-liver-brain interactions in tissue damage and disease development. *World Journal of Gastroenterology*.

[2] Shield K. D., Parry C., Rehm J. (2013). Chronic diseases and conditions related to alcohol use. *Alcohol Research*.

[3] Vassallo G., Mirijello A., Ferrulli A. (2015). Review article: alcohol and gut microbiota—the possible role of gut microbiota modulation in the treatment of alcoholic liver disease. *Alimentary Pharmacology and Therapeutics*.

[4] Leclercq S., Cani P. D., Neyrinck A. M. (2012). Role of intestinal permeability and inflammation in the biological and behavioral control of alcohol-dependent subjects. *Brain, Behavior, and Immunity*.

[5] Beutler B., Du X., Poltorak A. (2001). Identification of toll-like receptor 4 (Tlr4) as the sole conduit for LPS signal transduction: genetic and evolutionary studies. *Journal of Endotoxin Research*.

[6] Muller J. M., Ziegler-Heitbrock H. W. L., Baeuerle P. A. (1993). Nuclear factor kappa B, a mediator of lipopolysaccharide effects. *Immunobiology*.

[7] Afshar M., Richards S., Mann D. (2015). Acute immunomodulatory effects of binge alcohol ingestion. *Alcohol*.

[8] Bala S., Marcos M., Gattu A., Catalano D., Szabo G. (2014). Acute binge drinking increases serum endotoxin and bacterial DNA levels in healthy individuals. *PLoS ONE*.

[9] Tiwari V., Kuhad A., Chopra K. (2009). Suppression of neuro-inflammatory signaling cascade by tocotrienol can prevent chronic alcohol-induced cognitive dysfunction in rats. *Behavioural Brain Research*.

[10] Valles S. L., Blanco A. M., Azorin I. (2003). Chronic ethanol consumption enhances interleukin-1-mediated signal transduction in rat liver and in cultured hepatocytes. *Alcoholism: Clinical and Experimental Research*.

[11] Suryanarayanan A., Carter J., Landin J., Morrow A., Werner D., Spigelman I. (2016). Role of interleukin-10 (IL-10) in regulation of GABAergic transmission and acute response to ethanol. *Neuropharmacology*.

[12] Qin L., He J., Hanes R. N., Pluzarev O., Hong J.-S., Crews F. T. (2008). Increased systemic and brain cytokine production and neuroinflammation by endotoxin following ethanol treatment. *Journal of Neuroinflammation*.

[13] Qin L., Wu X., Block M. L. (2007). Systemic LPS causes chronic neuroinflammation and progressive neurodegeneration. *GLIA*.

[14] Barr T., Helms C., Grant K., Messaoudi I. (2016). Opposing effects of alcohol on the immune system. *Progress in Neuro-Psychopharmacology & Biological Psychiatry*.

[15] Neupane S. P., Lien L., Martinez P. (2014). High frequency and intensity of drinking may attenuate increased inflammatory cytokine levels of major depression in alcohol-use disorders. *CNS Neuroscience and Therapeutics*.

[16] Saunders J. B., Aasland O. G., Babor T. F., De La Fuente J. R., Grant M. (1993). Development of the Alcohol Use Disorders Identification Test (AUDIT): WHO collaborative project on early detection of persons with harmful alcohol consumption—II. *Addiction*.

[17] Jolliffe I. (2014). *Principal Component Analysis*.

[18] Szabo G., Saha B. (2015). Alcohol’s effect on host defense. *Alcohol Research: Current Reviews*.

[19] Kelley K. W., Dantzer R. (2011). Alcoholism and inflammation: neuroimmunology of behavioral and mood disorders. *Brain, Behavior, and Immunity*.

[20] Blednov Y. A., Ponomarev I., Geil C., Bergeson S., Koob G. F., Harris R. A. (2012). Neuroimmune regulation of alcohol consumption: behavioral validation of genes obtained from genomic studies. *Addiction Biology*.

[21] Harper K. M., Knapp D. J., Breese G. R. (2015). Withdrawal from chronic alcohol induces a unique CCL2 mRNA increase in adolescent but not adult brain—relationship to blood alcohol levels and seizures. *Alcoholism: Clinical & Experimental Research*.

[22] Na T.-Y., Han Y.-H., Ka N.-L. (2015). 22-S-Hydroxycholesterol protects against ethanol-induced liver injury by blocking the auto/paracrine activation of MCP-1 mediated by LXR*α*. *Journal of Pathology*.

[23] He J., Crews F. T. (2008). Increased MCP-1 and microglia in various regions of the human alcoholic brain. *Experimental Neurology*.

[24] June H. L., Liu J., Warnock K. T. (2015). CRF-amplified neuronal TLR4/MCP-1 signaling regulates alcohol self-administration. *Neuropsychopharmacology*.

[25] Altara R., Manca M., Hermans K. C. M. (2015). Diurnal rhythms of serum and plasma cytokine profiles in healthy elderly individuals assessed using membrane based multiplexed immunoassay. *Journal of Translational Medicine*.

[26] Petrovsky N., McNair P., Harrison L. C. (1998). Diurnal rhythms of pro-inflammatory cytokines: regulation by plasma cortisol and therapeutic implications. *Cytokine*.

[27] Pascual M., Baliño P., Aragón C. M. G., Guerri C. (2015). Cytokines and chemokines as biomarkers of ethanol-induced neuroinflammation and anxiety-related behavior: role of TLR4 and TLR2. *Neuropharmacology*.

[28] Tiwari V., Kuhad A., Chopra K. (2011). Amelioration of functional, biochemical and molecular deficits by epigallocatechin gallate in experimental model of alcoholic neuropathy. *European Journal of Pain*.

[29] Neupane S. P., Skulberg A., Skulberg K. R., Aass H. C. D., Bramness J. G. (2016). Cytokine level changes following acute ethanol intoxication in healthy men: a cross-over study. *Brain, Behavior, and Immunity*.

